# High mobilization of CD133+/CD34+ cells expressing HIF-1α and SDF-1α in septic abdominal surgical patients

**DOI:** 10.1186/s12871-020-01068-w

**Published:** 2020-06-27

**Authors:** Antonella Cotoia, Olga Cela, Gaetano Palumbo, Sabrina Altamura, Flavia Marchese, Nicoletta Mangialetto, Daniela La Bella, Vincenzo Lizzi, Nazzareno Capitanio, Gilda Cinnella

**Affiliations:** 1 Department of Anesthesia and Intensive care, University Hospital of Foggia, Foggia, Italy; 2 Department of Clinical and Experimental Medicine, University of Foggia, Foggia, Italy; 3 Hematology and BMT Unit, University Hospital of Foggia, Foggia, Italy; 4 Department of Medical and Surgical Science University Hospital of Foggia, Foggia, Italy

**Keywords:** Endothelial progenitor cells, Hypoxia inducible factor-1α, Stromal cell-derived factor-1α, Sepsis, Postoperative abdominal laparoscopic patients, Hematopoietic stem cells

## Abstract

**Background:**

The control of endothelial progenitor cells (CD133+/CD34+ EPCs) migrating from bone marrow to peripheral blood is not completely understood. Emerging evidence suggests that stromal cell-derived factor-1α (SDF-1α) mediates egression of EPCs from bone marrow, while the hypoxia inducible factor (HIF) transcriptional system regulates SDF-1α expression. Our study aimed to investigate the time course of circulating CD133+/CD34+ EPCs and its correlation with the expression of HIF-1α protein and SDF-1α in postoperative laparoscopic abdominal septic patients.

**Methods:**

Postoperative patients were divided in control (C group) and septic group (S group) operated immediately after the diagnosis of sepsis/septic shock. Blood samples were collected at baseline (0), 1, 3 and 7 postoperative days for CD133+/CD34+ EPCs count expressing or not the HIF-1α and SDF-1α analysis.

**Results:**

Thirty-two patients in S group and 39 in C group were analyzed. In C group CD133+/CD34+ EPCs count remained stable throughout the study period, increasing on day 7 (173 [0–421] /μl vs baseline: *P* = 0.04; vs day 1: *P* = 0.002). In S group CD133+/CD34+ EPCs count levels were higher on day 3 (vs day 1: *P* = 0.006 and day 7: *P* = 0.026). HIF-1α expressing CD133+/CD34+ EPCs count decreased on day 1 as compared with the other days in C group (day 0 vs 1: *P* = 0.003, days 3 and 7 vs 1: *P* = 0.008), while it was 321 [0–1418] /μl on day 3 (vs day 1; *P* = 0.004), and 400 [0–587] /μl on day 7 in S group. SDF-1α levels were higher not only on baseline but also on postoperative day 1 in S vs C group (219 [124–337] pg/ml vs 35 [27–325] pg/ml, respectively; *P* = 0.01).

**Conclusion:**

Our results indicate that sepsis in abdominal laparoscopic patients might constitute an additional trigger of the EPCs mobilization as compared with non-septic surgical patients. A larger mobilization of CD133+/CD34+ EPCs, preceded by enhanced plasmatic SDF-1α, occurs in septic surgical patients regardless of HIF-1α expression therein.

**Trial registration:**

ClinicalTrials.gov no. NCT02589535. Registered 28 October 2015.

## Background

The abdomen (post-operative infection, perforation, anastomotic leak) is the second most common site of sepsis and septic shock in Intensive Care Unit (ICU) [1–6]. Sepsis pathophysiology can be schematically described as an early hyper-inflammatory phase followed by a late hypo-inflammatory and immunosuppressive phase [7]. In addition, loss of endothelial barrier function, inflammation and impaired cellular oxygen delivery have been shown to be primary contributor to sepsis-related organ dysfunction [8].

Endothelial progenitor cells (EPCs) are immature hematopoietic stem cells which share the same precursor within bone marrow (BM) and are able to induce endothelial differentiation in peripheral blood (PB) [9–12]. Mutunga et al. observed an increase of circulating EPCs in sepsis and concluded that endothelial damage occurs [13]. Conversely, the group of Cribbs showed that EPCs were lower in septic patients compared with ICU patients controls and healthy controls [14]. To date, little is known about the linkage between EPCs and septic postoperative abdominal laparoscopic patients.

During the past two decades, the term ‘EPCs’ has been used to identify several types of cells belonging to different stages of differentiations into mature endothelial cells. The EPCs phenotype is common between hematopoietic stem cells and differentiated endothelial cells and a unique EPCs surface marker is still undetermined. However, it is commonly accepted that EPCs, in BM or immediately circulating, express CD133+/CD34+/ VEGFR2 [15].

Furthermore, the pathway of EPCs migrating from BM to PB has not been completely understood. Emerging evidence suggests that stromal cell-derived factor-1a (SDF-1α), a small cytokine belonging to the chemokine family, promotes the chemotactic EPCs migration from BM to PB, while the hypoxia inducible factor (HIF) transcriptional system regulates the SDF-1α expression, and thus it is considered a master mediator of the cellular adaptation to hypoxic microenvironments [16]. The importance of HIF to face hypoxia both at the cellular and organismal level has been recently recognized by the Nobel Prize in Physiology and Medicine 2019 [17].

Interestingly, it has been shown that CD133+/CD34+ hematopoietic stem/progenitor cells express high levels of stable extra-nuclear cytoplasmic form of the HIF-1α protein under normoxic conditions and proposed that SDF-1 expression is an early event in subsequent signal transduction pathways [18].

Our study aimed to investigate the time course of circulating CD133+/CD34+ EPCs either expressing or not the HIF-1α protein and its correlation with the plasma levels of SDF-1α in postoperative abdominal sepsis.

## Methods

This study was designed to target septic patients undergoing laparoscopic major abdominal surgery at University Hospital of Foggia, in an attempt to apply a study population as homogeneous as possible and to reduce confounding variables occurring when patients with different causes of sepsis are recruited.

After approval of the local research Ethics Committee (Comitato Etico of Ospedali Riuniti, Foggia, Italy, 69/CE/2015) and written informed consent obtained by each patients, the study was performed from January 2016 to December 2018 (ClinicalTrials.gov: NCT02589535).

Inclusion criteria were: age > 18 years old Caucasian patients, laparoscopic colon surgery under general anesthesia. Exclusion criteria were: metastatic cancer, palliative care, organ transplant and pregnancy because have been shown higher levels of EPCs or HIF [19–21].

### Intraoperative procedures

After intravenous cannula insertion, routine physiologic monitoring was applied, including temperature (SpotOn™ Temperature Monitoring System Model 370, MN, USA), neuromuscular function at the adductor pollicis (TOFWatch SXⓇ,Organon Teknik, Dublin, Ireland), dept. of anesthesia by bi-spectral index (BIS) monitoring (Aspect A-2000®; Aspect Medical System, Newton, MA).

Anesthesia was induced with propofol 2 mg/kg, fentanyl 3 μcg /kg and was maintained with an infusion of propofol 200 mg·kg^− 1^·min^− 1^, remifentanil 0.1–0.2 μcg·kg^− 1^·min^− 1^ and cisatracurium 1.5 mg·kg^− 1^·min^− 1^. The infusion rate of propofol varied in order to maintain a BIS value as close as possible to 50 and between 40 and 60 [22]. Patients were mechanically ventilated with a Servo Ventilator 900C (Siemens-Elema AB, Berlin, Germany) with 40% inspired oxygen, 5 cm H_2_O positive end-expiratory pressure, tidal volume (Vt) of < 8 ml/kg predicted body weight, 1:2 of I:E ratio. The respiratory rate was adjusted to reach normocapnia [23]. Postoperative patients enrolled in the study were divided into two groups:

-Control group (C group): non septic patients admitted to the surgical ward.

-Septic group (S group): septic patients admitted to the ICU, operated immediately after the diagnosis of sepsis/septic shock and treated with standard conventional therapy according to the Third International Consensus Definitions for Sepsis and Septic Shock [24].

Demographic data were obtained preoperatively, while clinical and laboratory data were assessed daily, according to routine procedure in the surgical ward and ICU. Mortality was defined as death occurring within 28 days after the diagnosis.

For this study, clinical and laboratory data were recorded at the following time points:

- baseline (0), 1, 3 and 7 postoperative days both in S and C groups.

Peripheral whole blood samples were utilized for flow cytometric determination of CD133+/CD34+ EPCs and HIF-1α, and the plasma samples were collected and stored at − 80 °C for SDF-1α analysis.

Differently from the study protocol (ClinicalTrials.gov: NCT02589535), voluntary healthy people and the postoperative day 10 in C group were excluded for the difficulty to obtain the blood samples; patients treated by extracorporeal hemoperfusion therapy in S group were excluded due to the difficulty on obtaining blood samples at the fixed time points since the treatment decision was made by the physician in charge at the moment considering the clinical situation and endotoxin plasma levels.

### Flow cytometry

As previously described [25], whole blood samples (100 μl) were treated within 1 h from withdrawal. For the viability analysis, cells were stained with 7-aminoactinomycin (7-AAD), which only binds to the DNA of those cells undergoing or have already undergone apoptosis (Stem Kit™ Beckman Coulter, Brea, CA, USA). Cell surface immuno-staining was performed according to the manufacturer’s guidelines adding the following fluorochrome-labeled monoclonal antibodies: fluorescein isothiocyanate (FITC)-conjugated anti-CD34 (Beckman-Coulter, Brea, CA, USA), phycoerythrin (PE)-conjugated anti-CD133 (Miltenyi Biotech, USA), peridinin chlorophyll protein complex (PerCP)-conjugated anti-human CD45 (Beckman-Coulter, Brea, CA, USA), PE-conjugated anti-human CD31 (Beckman-Coulter, Brea, CA, USA). Cells were fixed and permeabilized using the BD Intrasure kit (Becton Dickinson, Franklin Lakes, NJ, USA) and intracellular staining of HIF-1α was performed using Alexa Fluor® 647 anti-human HIF1α antibody (BioLegend Inc., CA, USA) following the manufacturer’s instructions. All samples were re-suspended in phosphate-buffered saline, and analysed by the use of a FACS Calibur flow cytometer (Becton Dickinson, Franklin Lakes, NJ, USA) and the FCS3 software. Cells were acquired with a four-parameter flow cytometry method (CD34 FITC/CD133 PE/CD45 PerCP/ HIF-1α ACP staining, side and forward angle light scatter). The flow cytometer was calibrated every 24 h and quality controls were performed according to the UK National External Quality Assessment Service (NEQAS) protocol. The number of CD133/CD34 cells expressing HIF-1α in peripheral blood was calculated on the basis of absolute lymphocytes count x percentage (%) of gated CD133/CD34 positive, and expressed as absolute number of cells per 1.0 μl peripheral blood.

### Enzyme-linked immunosorbent assay (ELISA)

Plasma concentration of SDF-1α was assessed by the enzyme-linked immunosorbent assay kit (SDF-1α Human ELISA kit, Abcam, It) according to the manufacturer’s instructions [25].

### Statistical analysis

Power analysis suggested that a sample size of 30 patients/group was required to detect a higher percentage of EPCs (CD133+/CD34+ cells in the percentage of all myelomonocytic cells) in septic patients vs ICU controls (mean ± standard error of mean: 0.52% ± 0.4% vs 0.24% ± 0.11%, respectively), assuming α = .05 and power = .95 [26]. This number was increased to 33 per group to allow for a 20% patients drop-out rate.

The normality of distribution was assessed by Shapiro-Wilkinson test. Data were expressed as mean ± SD, median [25–75] as appropriate. They were analyzed using repeated measurements analysis of variance. Differences between the groups at each time point were examined post hoc using independent sample t-test. A paired sample t-test was used to detect changes within the groups. Level of statistical significance was chosen at *P* < 0.05. Correlation analysis was performed by Spearman’s correlation analysis. Statistical analysis was performed by Statistical Package for the Social Sciences (SPSS Inc., Chicago, IL) version 15.0 for Windows.

## Results

**Fig. 1:**
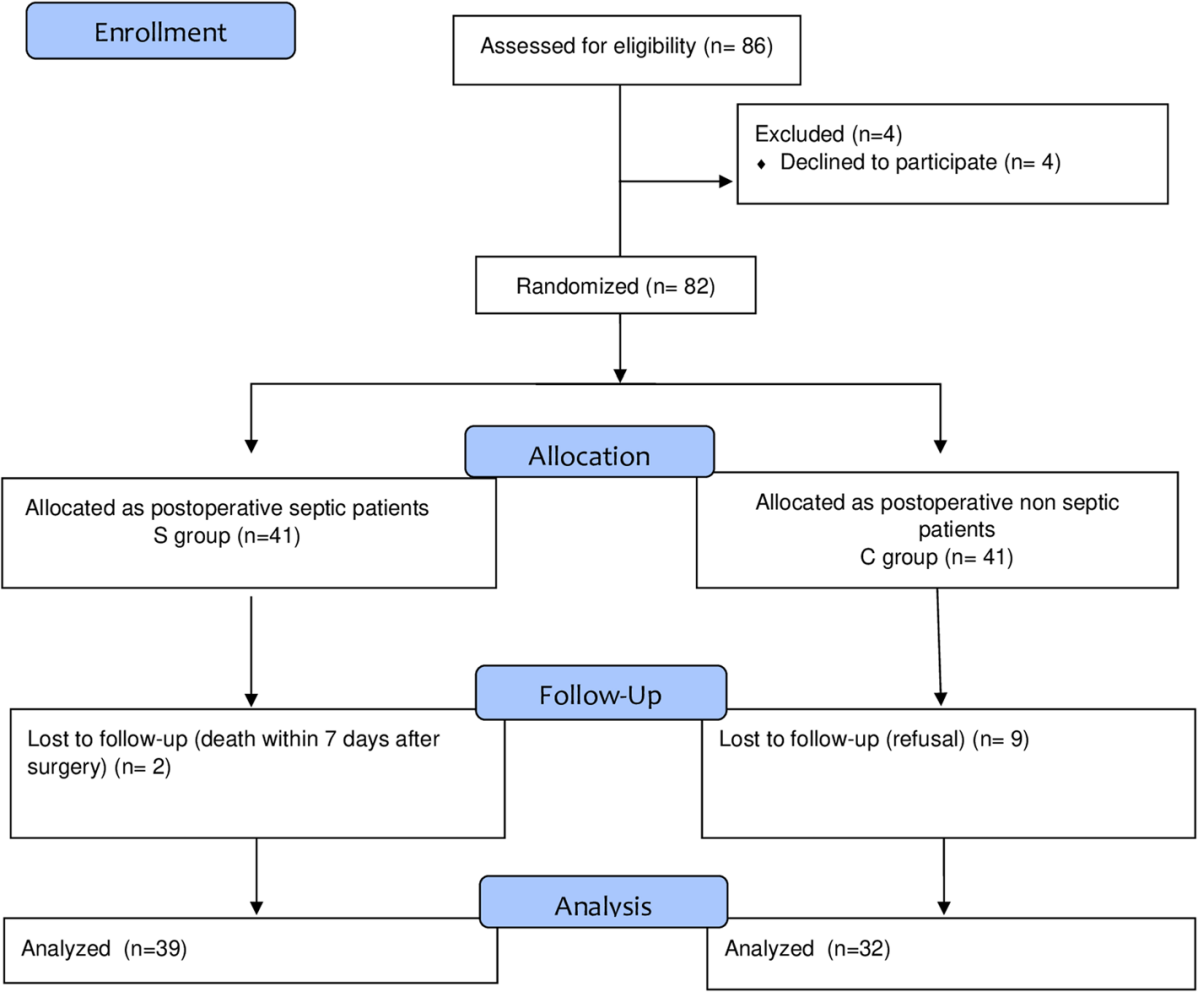
Flowchart of patients’ enrollment

The enrollment flow diagram is reported in Fig. 1. Seventy-one out of 82 candidates for enrollment were analyzed: 32 in S group and 39 in C group.

The demographic data were similar between the S and C groups. Clinical and laboratory data showed significant differences between S and C group at baseline (Table 1).

**Table 1:** Demographic, clinical and laboratory data at baseline

	Control (*n* = 39)	Sepsis (*n* = 32)	*P*-value
Age (years)	67 ± 11	70 ± 11	0.95
M/F	18/14 (56/44%)	24/15 (61/39%)	0.37
BMI (kg/m^2^)	28.53 ± 3.11	30.66 ± 5.63	0.17
APACHE II	8.87 ± 3.8	22.51 ± 7.74	< 0.001
SOFA score	4 ± 2.18	14.44 ± 7.75	< 0.001
Laparoscopic hemicolectomy elective/emergency,n (%)	27/4 (87/13%)	9/30 (23/77%)	< 0.001
Serum lactate (mmol/L)	0.73 ± 0.47	3.04 ± 2.61	< 0.001
Mechanical ventilation, n (%)	0 (0%)	31 (80%)	< 0.001
P/F	345 ± 78.73	250 ± 125.23	0.002
Vasoactive drugs, n (%)	0 (0%)	29 (75%)	< 0.001
Plasma procalcitonin (mcg/L)	0.85 ± 1.55	40.12 ± 48.81	< 0.001
Plasma endotoxin (pg/ml)	0.36 ± 0.13	0.58 ± 0.15	< 0.001
White blood cells count (× 10^9^/L)	8.28 ± 3.39	11.44 ± 6.51	0.012
Hemoglobin (mg/dl)	11.6 ± 1.8	10.2 ± 1.8	0.005
Hematocrit (%)	33 ± 7	31 ± 5	0.3

Data are shown as mean ± SD, BMI: Body Mass Index

Ten patients out of 32 in S group died within 28-days of admission in ICU and all patients survived in C group.

Baseline CD133+/CD34+ cells count was 56 [0–150]/μl in C group and 131 [0–968]/μl in S group; *P* = 0.31. HIF-1α-expressing CD133+/CD34+ cells count was 43 [0–132]/μl in C group and 8 [0–742]/μl in S patients; *P* = 0.4 Baseline SDF-1α level was 30 [25–295]pg/ml in C group and 200 [78–246]pg/ml in S group; *P* = 0.03.

**Fig. 2:**
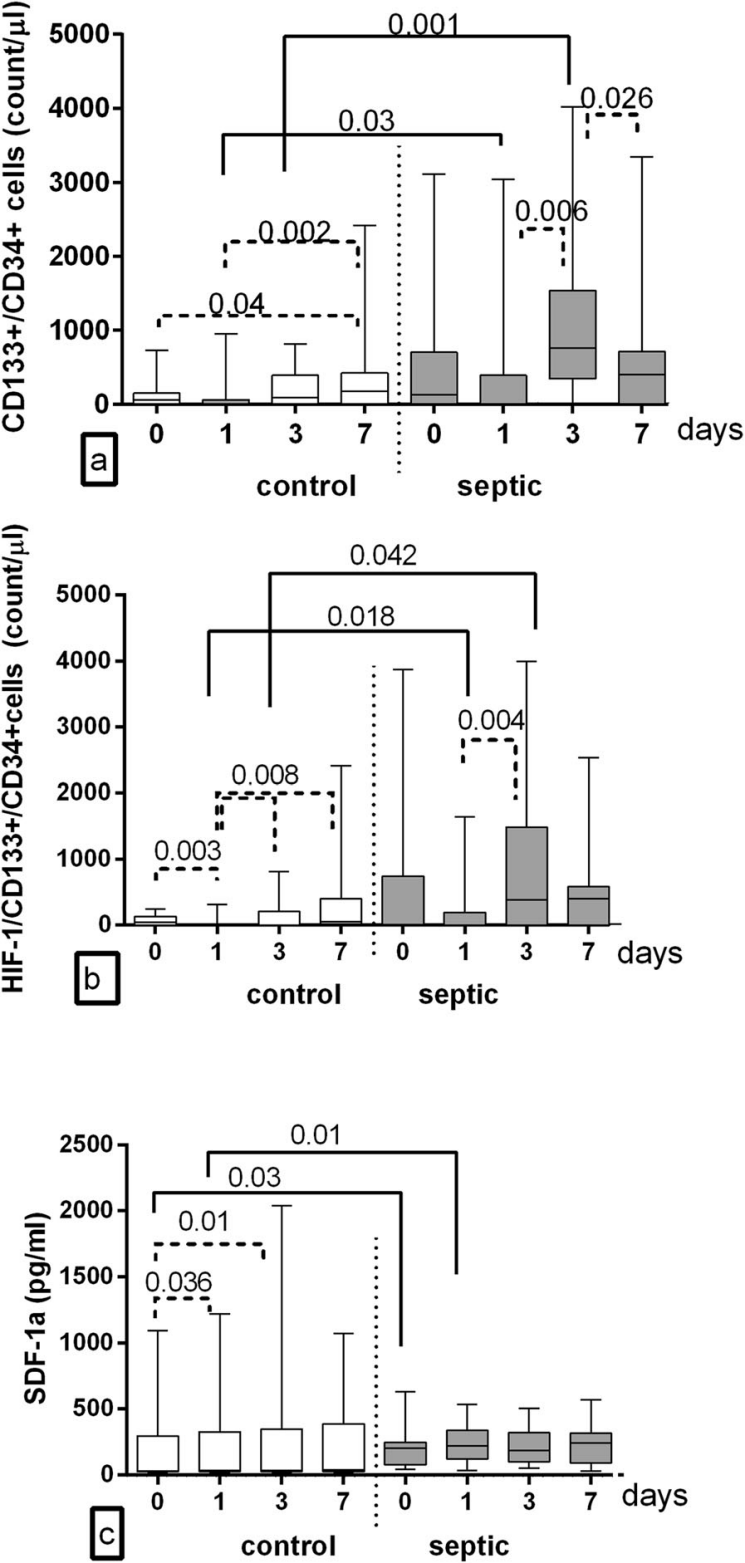
Statistical analysis of circulating CD133+/CD34+ cells (**a**), HIF-1α/CD133+/CD34 + cells (**b**) and SDF-1α level (**c**) at baseline and 1,3,7 postoperative days. SDF-1α, stromal derived factor 1 alpha; HIF-1α, hypoxia inducible factor 1 alpha. Data are shown as median [25–75]

Intragroup analysis in C patients showed that CD133+/CD34+ cell count remained stable throughout the whole study period, increasing on day 7 (173 [0–421] /μl vs baseline: *p* = 0.04; vs day 1: *P* = 0.002). In S group we observed higher CD133+/CD34+ cell count levels on day 3 (vs day 1: *P* = 0.006 and day 7: *P* = 0.026) (Fig. 2a).

HIF-1α-expressing CD133+/CD34+ cell count decreased on day 1 as compared with the other days in C group (day 0 vs 1: *P* = 0.003, days 3 and 7 vs 1: *P* = 0.008). In S group CD133+/CD34+ cell count expressing HIF-1α was 321 [0–1418] /μl on day 3 (vs day 1; *P* = 0.004), and 400 [0–587] /μl on day 7 (Fig. 2b).

SDF-1α levels significantly increased in C group on days 1 (vs baseline: *P* = 0.036) and day 3 (35 [28–346]pg/ml vs baseline: *p* = 0.01). No differences in SDF1-α levels were observed in S group throughout the whole study period (*P* > 0.19) (Fig. 2c).

Intergroup analysis showed that S group had higher CD133+/CD34+ cell count on days 1 and 3, (S vs C group at day 1: 0 [0–393] /μl vs 0 [0–44] /μl; P = 0.03 and day 3: 784 [264–1818] /μl vs 0 [0–43] /μl,; *P* = 0.001). HIF-1α-expressing CD133+/CD34+ cells count was higher in S vs C group throughout the whole study period, especially on 1 and 3 postoperative days (day 1: 0 [0–175]/μl vs 0 [0–1]/μl, respectively; *P* = 0.018 and day 3: 321 [0–1418]/μl vs 0 [0–208]/μl; *P* = 0.042). SDF-1α levels were higher not only on baseline but also on postoperative day 1 in S vs C group (219 [124–337] pg/ml vs 35 [27–325] pg/ml, respectively; P = 0.01) (Figs. 2a-c).

**Fig. 3:**
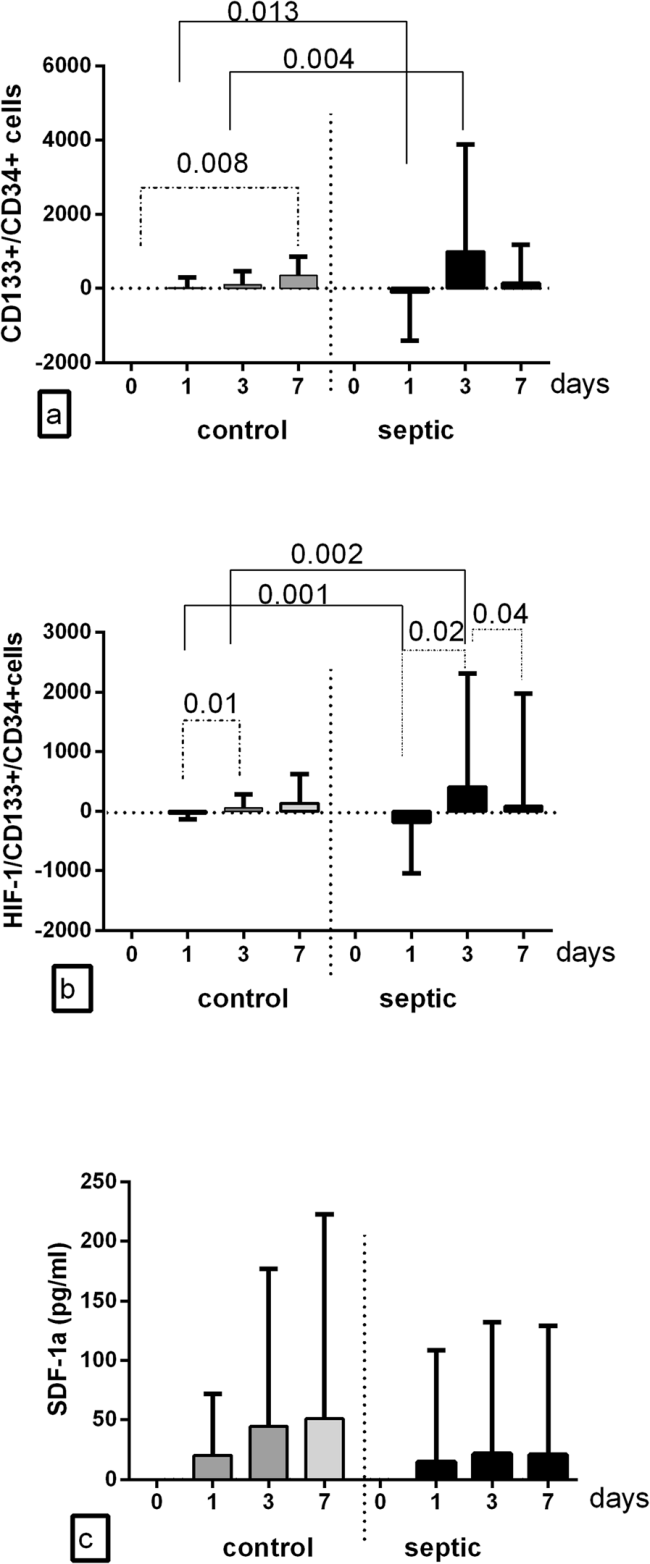
Circulating CD133+/CD34+ cells **(a**), HIF-1α/CD133+/CD34 + cells (**b**) and SDF-1α level (**c**) levels at and 1,3,7 postoperative days were normalized to the baseline values of each subject. SDF-1α, stromal derived factor 1 alpha; HIF-1α, hypoxia inducible factor 1 alpha. Data are shown as mean ± SD

Comparable results were attained when the total circulating CD133+/CD34+ EPC and HIF-1α-expressing CD133+/CD34+ EPC and the SDF-1α content were normalized to the baseline values of each subject (Figs. 3 a-c).

No correlation was observed between lactate levels and SDF-1α (R = 0.42; *P* = 0.56) or HIF-1α- expressing CD133+/CD34+ cell count (R = 0.01; *P* = 0.85). Alike no correlation was found between SDF-1α or CD133+/CD34+ cells expressing HIF-1α and APACHE II or SOFA score and 28-days mortality. Although not statistically significant, likely because of the limited number of cases, a trend was observed showing a higher maintenance of the circulating CD133+/CD34+ EPC at day 3 in surviving septic patients compared to non-surviving (data not shown).

## Discussion

The main result of the present study is that, as compared with surgical controls, septic patients exhibit: i) a higher number of circulating CD133+/CD34+ cells, encompassing those expressing HIF-1α (CD133+/CD34 + -HIF-1α cells); ii) a higher plasmatic level of SDF-1α that does not correlate with plasma lactate.

To the best of our knowledge, this is the first study simultaneously investigating the kinetics of the CD133+/CD34+ cells, with or without HIF-1α and the plasmatic SDF-1α in septic patients. Furthermore, a novelty of our study is the evaluation of the EPCs in postoperative septic patients compared with laparoscopic controls. Indeed, though it is well established that surgical injury elicits mobilization of EPC, all the available studies focused on laparotomic surgery and no data are available in laparoscopic surgery which has now become the gold standard in colectomy [12, 27, 28].

Data in literature showed that circulating progenitors are present at higher levels in septic patients when compared with healthy volunteers, especially at 72 h after the diagnosis of sepsis [27, 29]. Sepsis is associated with systemic microcirculation disruption, hemodynamic imbalance and poor peripheral oxygen uptake although oxygen delivery can be maintained pharmacologically [4]. Hypoxic conditions is known to promote activation of the HIF-1 transcriptional system thus enabling cell adaptation to limited environmental oxygen availability. HIF-1α is the subunit of HIF-1 complex, is considered as a reliable biomarker of cellular hypoxia. During hypoxic conditions, the HIF-1α stabilization occurs inhibiting its degradation thereby resulting in the accumulation of the protein within the nucleus where upon forming a heterodimer with the HIF-1β subunit binds to hypoxia responsive elements located at the regulatory regions of a number of genes. Conversely, under normoxic conditions HIF-1α is continuously generated and degraded while HIF-1β is constitutively expressed in the cells regardless of oxygen tension [30, 31].

It has become increasingly evident that hypoxia, does not occur only under stressful conditions, but it is normally present in tissular microenvironments, such as the human BM endosteum, where the hematopoietic niches are located [18, 21, 31]. In this hypoxic environment the hematopoietic stem/progenitor cells largely rely on glycolysis to limit production of reactive oxygen species (ROS) thus preventing DNA oxidative damage [21, 31]. In the present study we succeeded in detecting HIF-1α in a subset of CD133+/CD34+ EPCs from freshly drawn blood and showed a higher number of them at days 1 and 3 of postoperative in septic patients vs controls.

Whereas it has been reported that HIF-1α expression was significantly suppressed in sepsis [31], several data convincingly showed that circulating EPCs levels in septic patients are elevated [32, 33]. An increased expression of stable HIF-1α in patients with shock, as compared with healthy volunteers during the 4 h of the study period from the diagnosis has been reported. However, the changes in HIF-1α expression over time were not correlated with the patient outcome [33]. Importantly, the EPCs functional competence in terms of regenerative capacity (i.e. their quality instead of quantity) appears to represent the “crux of the problem” in sepsis [9].

Interestingly, we report no correlation between plasma lactate and CD133+/CD34+ cells levels expressing HIF-1α, or plasmatic SDF-1α. Plasma lactate which represents the end product of glycolysis, is used as a marker of tissue hypoxia in sepsis and septic shock [24]. Therefore, the debate of considering the HIF-1a as a marker of the true level of tissue oxygenation is still open. However, lactate kinetics represents a balance between lactate production and clearance, which can hamper for the interpretation of cell hypoxia in septic shock at the bedside [34].

**Fig. 4:**
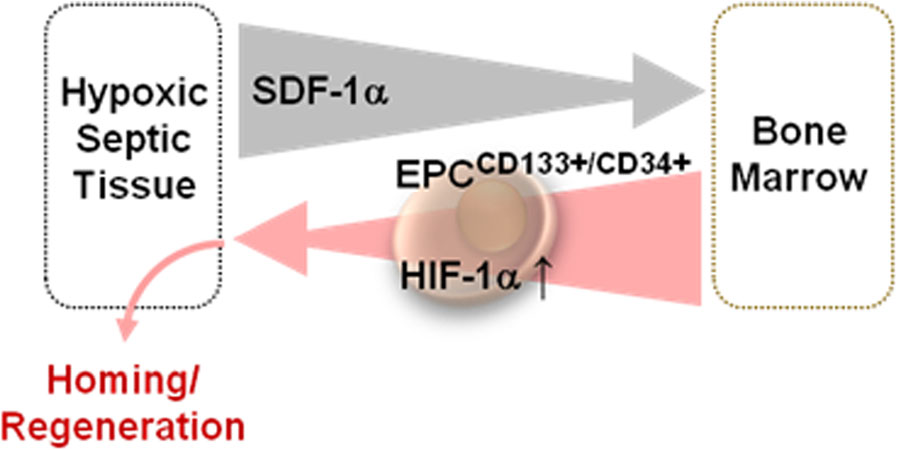
Proposed mechanism of the SDF-1α-mediated mobilization of EPC (CD133+/CD34+) in septic patients. SDF-1α, stromal derived factor 1 alpha; EPC, endothelial progenitor cell; HIF-1α, hypoxia inducible factor 1 alpha. See Discussion for details

A possible explanation reconciling conflicting results is provided by Piccoli and al., whose results suggest that the chemokine-dependent mobilization from the BM is a trigger for the stabilization of HIF-1α under normoxic conditions in healthy donors. Therefore, the initial leading event could be linked to the chemoattractant SDF-1α, which promotes HIF-1α stabilization in mobilized endothelial/hematopoietic stem cells [18]. This might constitute a pre-conditioning mechanism promoting EPC adaptation to hypoxic injured septic districts, thus preserving their regenerative capacity (Fig. 4).

In view of the above proposed mechanism we might explain the observed higher SDF-1α levels on baseline and 1 postoperative day followed by the chemotactic recruitment of CD133+/CD34+ cells expressing stable HIF-1α in the succeeding observational period in surgical septic patients. To note, the ratio of the HIF-1α-expressing CD133+/CD34+ EPC/total CD133+/CD34+ EPC does not change irrespective both of the sampling (i.e. control vs septic patients) and of the time points (data not shown). This would imply that the expression of HIF-1α, even under normoxia, is an intrinsic feature of EPCs not triggered per se by SDF-1α, resembling what previously reported for CD34+ hematopoietic stem/progenitor cells [18].

In the perspective of potential therapeutic strategies with EPCs in combination with SDF-1α analogue, which have been successfully administered in septic animal models, our findings represent a compelling reason to further study the kinetics of CD133+/CD34+ expressing stable HIF-1α and their linkage to SDF-1α in human sepsis [35].

A potential limitation of our study is that our EPC population counts likely includes small amounts of hematopoietic stem cells, since the classical definition of EPCs requires an endothelial marker protein like VEGF-R2 or CD31 [36]. However, we counted CD31/CD45 cells (data not shown) by using the anti-CD31 in our flow cytometry analysis, and their number was repetitively equal to the CD133+/CD34+ cells count.

## Conclusions

Although further studies are needed the data here presented show for the first time a larger mobilization of HIF-1α expressing CD133+/CD34+ cells preceded by plasmatic SDF-1α in septic surgical patients regardless of tissue oxygenation.

## Data Availability

The datasets used and/or analyzed during the current study are available from the corresponding author on reasonable request.

## Abbreviations

ICU: Intensive Care Unit
EPCs: Endothelial progenitor cells
BM: Bone marrow
SDF-1α: Stromal cell-derived factor-1a
HIF-1α: Hypoxia inducible factor-1α
